# Association of Microglial Activation With Spontaneous ARIA-E and CSF Levels of Anti-Aβ Autoantibodies

**DOI:** 10.1212/WNL.0000000000200892

**Published:** 2022-09-20

**Authors:** Fabrizio Piazza, Silvia Paola Caminiti, Marialuisa Zedde, Luca Presotto, Jacopo C. DiFrancesco, Rosario Pascarella, Alessia Giossi, Maria Sessa, Loris Poli, Gianpaolo Basso, Daniela Perani

**Affiliations:** From the CAA and AD Translational Research and Biomarkers Laboratory (F.P.), School of Medicine and Surgery (J.C.D., G.B.), iCAβ International Network (F.P., M.Z., J.C.D., A.G., M.S., Loris Poli, G.B.), and SINdem CAA Study Group (F.P., M.Z., D.P.), University of Milano-Bicocca, Monza; Vita-Salute San Raffaele University (S.P.C., D.P.), Milan; IRCCS San Raffaele Scientific Institute (S.P.C., Luca Presotto, D.P.), Milan; Neurology Unit (M.Z.), Stroke Unit, Azienda UnitÃ Sanitaria Locale–IRCCS di Reggio Emilia; Neuroradiology Unit (R.P.), Azienda UnitÃ Sanitaria Locale–IRCCS di Reggio Emilia; Neurology Unit (A.G.), Azienda Socio-Sanitaria Territoriale di Cremona; Neurology Unit (M.S.), Ospedale ASST Papa Giovanni XXIII, Bergamo; and Neurology Unit (Loris Poli), ASST Spedali Civili, Brescia, Italy.

## Abstract

**Background and Objectives:**

Amyloid-related imaging abnormalities suggestive of vasogenic edema or sulcal effusion (ARIA-E) are the most common adverse events complicating Alzheimer disease (AD) immunotherapy with anti–β-amyloid (Aβ) monoclonal antibodies. ARIA-E can also occur spontaneously in cerebral amyloid angiopathy–related inflammation (CAA-ri), a rare autoimmune encephalopathy associated with increased CSF levels of anti-Aβ autoantibodies. Although the pathophysiologic mechanisms of ARIA-E remain to be fully elucidated, experimental evidence from ex vivo studies suggests that gantenerumab and aducanumab enable microglial activation. However, the in vivo evidence for a direct association between neuroinflammation and ARIA-E in patients with high CSF anti-Aβ (auto)antibody levels has never been demonstrated.

**Methods:**

The spatial distribution and temporal variations of microglial activation associated with levels of anti-Aβ autoantibodies at (sub)acute presentation of ARIA-E and after corticosteroid therapy were evaluated in a longitudinal case series of patients with CAA-ri, the spontaneous variant of the iatrogenic ARIA-E reported in Aβ-lowering immunotherapy with monoclonal antibodies. Multimodal and multiparametric MRI was used for CAA and ARIA-E severity quantification, according to validated scoring system; CSF testing for anti-Aβ autoantibodies and AD biomarkers; _11_C-PK11195 PET for activated microglia.

**Results:**

At (sub)acute presentation, we found focal peaks of microglial activation having a greater spatial colocalization with ARIA-E compared with chronic age-related white matter change imaging abnormalities. The severity of ARIA-E and the magnitude of the associated microglial activation were greater in patients having AD and severe CAA concomitant disease compared with patients having CAA only. CSF anti-Aβ autoantibodies at presentation were high in all patients and markedly decreased at posttreatment follow-up, in parallel with clinical resolution of acute symptoms, reduced ARIA-E severity, and reduced microglial activation.

**Discussion:**

Our findings extend the current notion of ARIA-E by providing the first in vivo ^11^C-PK11195 PET evidence for an association between microglial activation and the magnitude and severity of ARIA-E in patients with increased CSF concentration of anti-Aβ autoantibodies and comorbid AD and CAA disease. Our results highlight CSF testing for anti-Aβ autoantibodies as a promising diagnostic, prognostic, and therapy response biomarker to help guide future treatment and management decisions in real clinical practice and clinical trials.

Cerebral amyloid angiopathy–related inflammation (CAA-ri) is a rare autoimmune encephalopathy associated with spontaneous symptomatic amyloid-related imaging abnormalities suggestive of vasogenic edema (ARIA-E) that are thought to be linked to an exaggerated autoantibody immune reaction against β-amyloid (Aβ) of CAA and Alzheimer disease (AD).^1,2^ Evidence from a large cohort registry study of inpatients with CAA-ri showed that the natural history of spontaneous ARIA-E shares striking clinical, radiologic, and biological similarities with the iatrogenic ARIA-E reported in up to 50% of patients with AD exposed to several anti-Aβ monoclonal antibodies (mAbs) tested in clinical trials.^1,3–5^

Clinically, CAA-ri presents with (sub)acute cognitive changes, seizures, focal neurologic deficits, and altered mental state. Radiologically, patients with CAA-ri present with MRI evidence of focal corticosubcortical hyperintensities suggestive of parenchymal vasogenic edema (VE) and/or sulcal effusion on T2-weighted fluid-attenuated inversion recovery (FLAIR) images (i.e., spontaneous ARIA-E).^1,6^ Both spontaneous and iatrogenic ARIA-E have a transient and potentially relapsing nature, with a suggested positive effect of corticosteroid therapy on prognosis and for the prevention of subsequent recurrences.^1,7,8^

According to currently available criteria, at least one of the following imaging markers is also required to make a diagnosis of CAA-ri: lobar cerebral microbleeds (CMBs), intracerebral hemorrhage (ICH), and cortical superficial siderosis (cSS) on gradient echo-T2*-weighted (GRE-T2*) images.^6^ Unlike ARIA-E, these MRI markers typically do not resolve at follow-up and the burden increases with disease progression.^3^

Biologically, CAA-ri is characterized by high levels of anti-Aβ autoantibodies in the CSF during the (sub)acute stage of the disease, returning within levels typically observed in patients with AD and noninflammatory CAA after clinicoradiologic resolution of ARIA-E.^2,9-14^ Based on the above evidence, ARIA-E of AD immunotherapy is increasingly recognized as an iatrogenic manifestation of the spontaneous ARIA-E associated with increased CSF anti-Aβ autoantibodies occurring in both patients with AD and CAA.^2,10,12,15,16^

The pathophysiologic mechanisms of ARIA-E remain to be fully elucidated. Experimental evidence from ex vivo studies suggests that the mAbs gantenerumab and aducanumab enable microglial activation.^17–19^ However, the in vivo evidence for a direct association between neuroinflammation and ARIA-E in patients with high CSF anti-Aβ (auto)antibody levels has never been demonstrated so far.

Given that microglia can either contribute to clearing Aβ by cell-mediated phagocytosis or increase the risk of ARIA-E by triggering an exaggerated neuroinflammatory response, elucidating the biology of the neuroinflammatory response associated with ARIA-E will be key for guiding future treatment decisions.^1,4,5,7,8,10,12,20,21^ Here, through a multimodal and multiparametric MRI, CSF, and PET study with ^11^C-PK11195, a tracer targeting the 18-kDa translocator-specific protein overexpressed in activated microglia cells,^22–24^ we describe the spatial distribution and temporal variations of in vivo microglial activation associated with ARIA-E at disease presentation and following corticosteroid therapy in a longitudinal case series of patients with well-defined CAA-ri.

## Methods

### Participants

We studied a case series of inpatients presenting with (sub)acute CAA-ri, diagnosed by clinical presentation, CSF testing, and neuroradiologic findings, according to the current criteria.^6^ Participants were prospectively enrolled throughout the iCAβ International Network longitudinal cohort registry of CAA-ri referred to the University of Milano-Bicocca (UNIMIB) coordinating center.^25^ The description of the multicenter, hospital-based, prospective, longitudinal cohort design has been previously reported.^1^

For this study, participants were selected based on the following eligibility criteria: (1) availability of MRIs and CSF samples collected at admission, before starting corticosteroids, (2) no contraindications to undergo a PET scan before starting treatment, (3) ability to travel to the Nuclear Medicine Unit at San Raffaele Hospital in Milan, and (4) availability to undergo a second PET scan and lumbar puncture at posttreatment follow-up monitoring. The first 4 consecutive patients who matched eligibility criteria and agreed to participate were enrolled.

Participants underwent baseline MRI, CSF, and PET within 3 months from symptom onset and before starting corticosteroid pulse therapy with 5 IV boluses of 1 g/d methylprednisolone for 5 consecutive days, with or without subsequent oral tapering-off. All procedures were repeated at posttreatment monitoring, assessed from ≥3 to 12 months from symptom onset. The therapeutic and follow-up monitoring schedule was defined according to the iCAβ International Network recommendations as described elsewhere.^1^ We followed the Strengthening the Reporting of Observational Studies in Epidemiology reporting guideline.

### MRI Acquisition

T1-weighted, GRE-T2*, susceptibility-weighted imaging, FLAIR, and diffusion-weighted imaging images were acquired on 1.5 T imaging systems, as previously described.^1^ MRI acquisitions followed imaging standard requirements proposed by the STandards for ReportIng Vascular changes on nEuroimaging working group.^26^ The reading of MRIs was centrally assessed by trained neuroradiologists (G.B. and R.P.), blinded to clinical and therapeutic data, as previously described.^1^

### ARIA-E Assessment

Semiautomated intensity-based drawing and editing tools (MRIcro v.1.0.19) were used to segment the region of interest (ROI) comprising the specific parenchymal and sulcal hyperintensities, which define ARIA-E (ARIA-E_roi) on baseline FLAIR images, according to the current definitions.^3^ The extent and radiologic severity of ARIA-E was quantified according to the validated 60-point Barkhof Grand Total Score (BGTS)^27^ as well as with the simplified ARIA-E severity score^28^ system currently adopted in AD clinical trials.

### CAA Load Assessment

Baseline T2*-GRE and FLAIR images were used to evaluate lobar CMB number,^29^ distribution and severity of cSS,^29^ number of centrum semiovale perivascular spaces, and deep and periventricular white matter hyperintensities of chronic cerebral small vessel disease (cSVD) and aging (globally defined as age-related white matter change [ARWMC]).^26^ All markers were rated with previously standardized and validated rating scores, according to the current consensus criteria and definitions.^29–33^ MRIcro v.1.0.19 editing tools were used to segment the ROI indicative of ARWMC (ARWMC_roi).

### Routine CSF and Serum Testing

According to the current CAA-ri criteria, all patients were tested for the absence of neoplastic, infectious, and other causes. CSF/serum albumin quotient (QAlb) and age-related maximum normal CSF/serum albumin (QNorm) were also calculated. According to Puthenparampil et al.,^34^ patients with QAlb > QNorm were considered as having increased blood-brain barrier permeability.

### CSF Anti-Aβ Autoantibodies, AT(N) Biomarker Profile, and ApoE Genotype

All tests were centrally assessed at the CAA and AD Translational Research and Biomarkers Laboratory of the University of Milano-Bicocca. The CSF level of anti-Aβ autoantibodies was quantified with an in-house immunoenzymatic bead-based ultrasensitive assay as previously described.^2^ CSF testing for Aβ42/Aβ40 ratio (A), p181-tau (T), and t-tau (N) was assessed with commercial ELISAs to determine the biomarker profile in the AD continuum, according to the AT(N) research framework criteria.^35,36^ Cutoff values were as follows: A(+) <0.1, T(+) ≥30 pg/mL, N(+) ≥400 pg/mL, and anti-Aβ autoantibodies(+) ≥32 ng/mL (borderline if ±10%). Values were settled according to the previously published data^2^ and current internal research programs.^25^ A detailed description of the methods used for CSF testing and ApoE genotyping can be found elsewhere.^2,37,38^

### ^11^C-PK11195 PET

^11^C-PK11195 PET scans were performed on a multiring PET Discovery 690 General Electric Medical System, injecting a dose of 380 ± 37 MBq of ^11^C-PK11195 with a radiochemical and chemical purity >95%. Acquisition protocol included a dynamic PET scan of 15 frames lasting 58 minutes (6 × 30 s/2 × 1 min/1 × 3 min/3 × 5 min/2 × 10 min/1 × 15 min). PET data were corrected for attenuation, radioactive decay, and scatter. For each scan, movement correction was executed by realigning individual frames over time using SPM5.^39^ Nuclear medicine experts, blinded to clinical and therapeutic data, centrally assessed the reading of PET images.

The change in ^11^C-PK11195 PET binding potentials (BPs) was estimated using a receptor parametric mapping procedure, requiring a preset reference region. Imaging data were analyzed using the curve distance clustering algorithm adaption of the validated SuperVised Clustering Algorithm to estimate the similarity of the time-activity curve (TAC) of each voxel with 4 predefined TACs (tracer delivery in blood, white matter, gray matter with nonspecific binding, and high specific binding).^40,41^

Each ^11^C-PK11195 PET BP was coregistered to the subject-specific FLAIR acquired at baseline. To remove nonspecific binding, each image was subtracted voxel by voxel for an average BP map obtained from an in-house data set of 10 healthy volunteers. Before comparison, the average BP map in the MNI space was propagated to each patient's native space by the inverse warping parameters identified through spatial normalization of the patient's FLAIR scan. All voxels with a BP ≥ 0.1, namely above the across-subjects BP cerebral global mean plus 1 SD, were considered BP peaks.

The percentage of spatial interaction between ^11^C-PK11195 PET BP peaks and each ROI (i.e., ARIA-E_roi and ARWMC_roi) was extracted considering the number of overlapping voxels normalized to the number of voxels of the ROI. Mean BP values extracted at baseline and at follow-up, within each ROI, were used to estimate posttreatment variations. For illustration purposes, single-patient delta reduction maps (ΔBP map) resulting from the voxel-vise subtraction of the baseline BP peaks from those at follow-up were also computed.

### Standard Protocol Approvals, Registrations, and Patient Consents

The UNIMIB Institutional Ethical Committee on human experimentations approved the study (biomarkARIA protocol #268/02112016 and modelCAA Protocol #372/16042018). The San Raffaele Hospital Institutional Ethical Committee approved the ^11^C-PK11195 PET study (Protocol #DSAN854-A/2). All patients provided written informed consent for the use of clinical, laboratory, and imaging data.

### Data Availability

The data that support the findings of this study are available from the corresponding authors on reasonable request.

## Results

### Case 1

A 76-year-old woman was admitted with focal epileptic seizure characterized by loss of contact with fixed gaze. The patient had a history of 2 lobar ICHs in the preceding 3 years, which led to the diagnosis of probable CAA.

**Figure 1:**
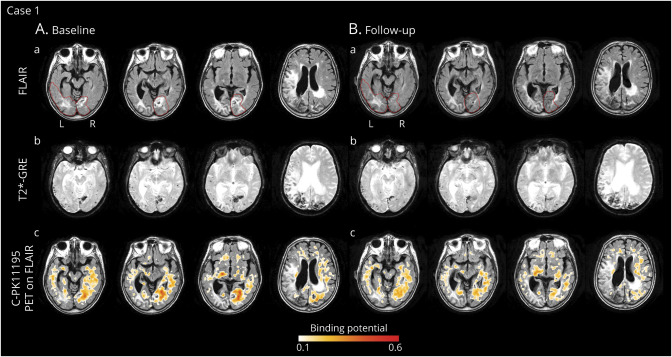
Longitudinal In Vivo Imaging of Microglial Activation During the Course of Spontaneous ARIA-E in a Patient With Probable CAA-ri (Case 1) (A) Baseline MRI and ^11^C-PK11195 PET images acquired within 1 week from presentation of (sub)acute symptoms. (a) T2-weighted (FLAIR) images showed spontaneous ARIA-E in the occipital lobe, bilaterally (red lines indicate the anatomic regions affected by ARIA-E). (b) Gradient echo-T2*-weighted imaging (GRE-T2*) sequence, coregistered to baseline FLAIR images, showed multiple cerebral microbleeds (CMBs) in the right and left occipital lobes, disseminated cortical superficial siderosis (cSS), and late subacute intracerebral hemorrhage (ICH) in the right occipital pole. CSF testing confirmed high concentrations of anti-Aβ autoantibodies.^2^ The diagnosis of probable CAA-ri was made.^6^ (c) 11C-PK11195 PET images, coregistered and superimposed onto baseline FLAIR images, revealed diffused binding potential peaks (BP peaks) of microglial activation, more evident within the ARIA-E anatomic region in the right occipital lobe. Treatment with high-dose corticosteroid pulse therapy was started, followed by slow tapering-off for the subsequent 5 months. (B) Follow-up MRI and ^11^C-PK11195 PET acquired 5 months after the starting of corticosteroids. MRI and PET images are coregistered to baseline FLAIR. (a) FLAIR images showed a marked reduction of vasogenic edema within the ARIA-E anatomic regions defined at baseline (red lines). (b) GRE-T2* images showed a reduction of the extent of signal drop near the subacute ICH in the right occipital pole and the appearing of 2 new CMBs localized outside the ARIA-E anatomic regions. (c) 11C-PK11195 PET revealed a globally reduced microglial activation that was more evident in the ARIA-E anatomic region. ARIA-E = amyloid-related imaging abnormalities suggestive of vasogenic edema; FLAIR = fluid-attenuated inversion recovery.

The same day, an MRI scan (Figure 1) showed severe ARIA-E in the occipital lobe, bilaterally, with a BGTS value of 7. T2*-GRE images showed >100 CMBs in the right and left occipital lobes, disseminated cSS, and late subacute ICH in the right occipital pole, corresponding to a CAA severity score of 5. The day after, CSF testing for anti-Aβ autoantibodies was positive.^2^ The AT(N) biomarker profile was A+T+(N)+, suggesting AD pathologic changes. ApoE genotyping showed ε3/ε3 allele carriage. All results are shown in Table.

**Table:**
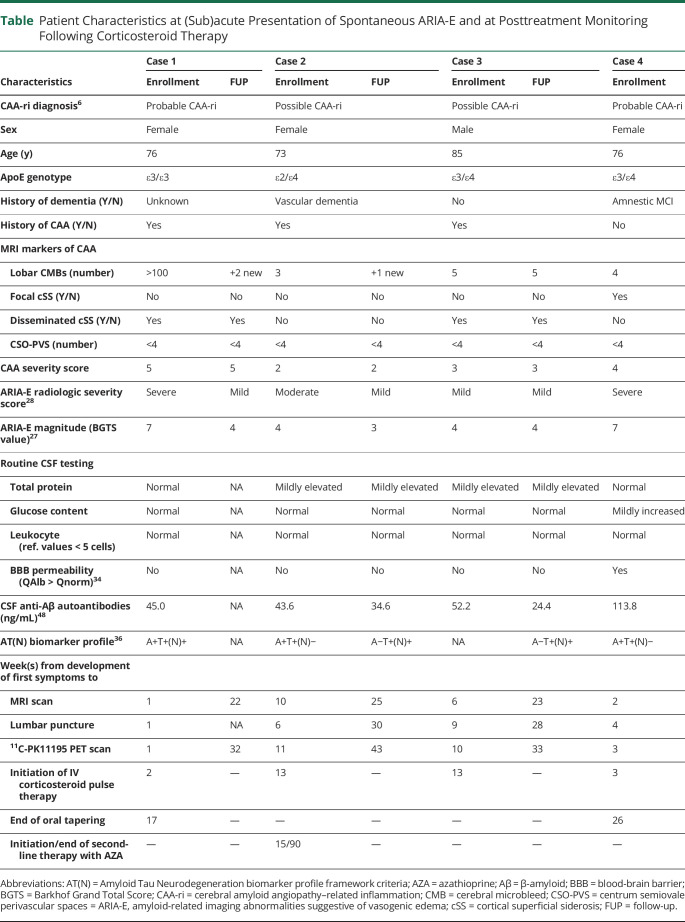
Patient Characteristics at (Sub)acute Presentation of Spontaneous ARIA-E and at Posttreatment Monitoring Following Corticosteroid Therapy

**Figure 2:**
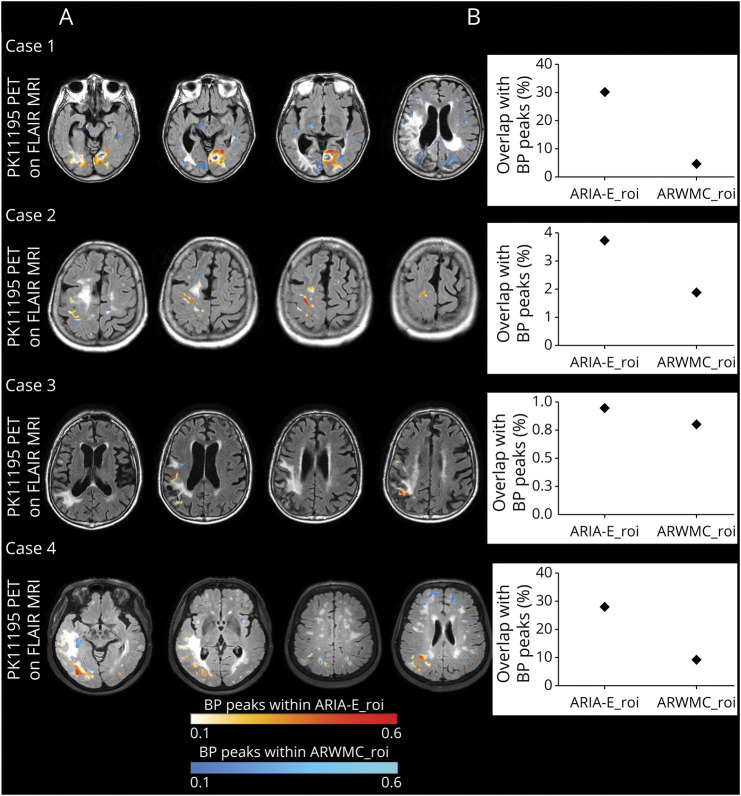
Microglial Activation Overlapping With ARIA-E and ARWMC at Baseline (A) The figure shows baseline ^11^C-PK11195 binding potential peaks (BP peaks) overlapping ARIA-E (ARIA-E_roi) and ARWMC (ARWMC_roi) regions of interest. For each patient, BP peaks were extracted from the same baseline BP peak maps displayed on the left lower rows of Figures 1–4 and superimposed on FLAIR images acquired at baseline. The intensity of microglial activation is represented using a yellow to red scale for BP peaks overlapping with ARIA-E_roi and a dark to light blue scale for BP peaks overlapping with ARWMC_roi. (B) Graphical representation of the percentage of spatial interaction between microglial activation and ARIA-E_roi and ARWMC_roi computed, respectively, as the total number of BP peaks and ROI overlapping voxels normalized to the number of voxels of the ROI. ARIA-E = amyloid-related imaging abnormalities suggestive of vasogenic edema; ARWMC = age-related white matter change; FLAIR = fluid-attenuated inversion recovery; ROI = region of interest.

According to clinical presentation, MRI, and CSF findings, a diagnosis of probable CAA-ri was made.^2^ Five days later, ^11^C-PK11195 PET revealed diffused BP peaks of microglial activation that were more evident within the ARIA-E anatomic region in the right occipital lobe (Figure 1). The spatial interaction of BP peaks with ARIA-E_roi and ARWMC_roi was 30.1% and 4.6%, respectively (Figure 2).

Four days after PET, treatment with IV high-dose corticosteroid pulse therapy was started, followed by 1 mg/kg of oral prednisolone and gradual tapering-off during the subsequent 5 months, with no recurrence of seizures. Five and a half months after admission, a second MRI scan (5 months after the starting of corticosteroids) showed a marked improvement of ARIA-E, with a 3-point reduction in the BGTS. T2*-GRE images revealed 2 new CMBs that were not localized within the anatomic regions affected by ARIA-E.

**Figure 3:**
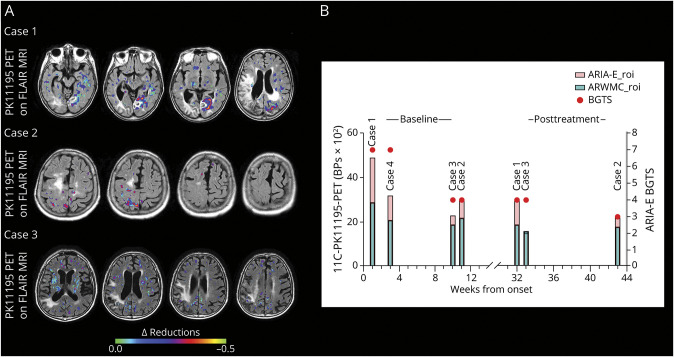
Delta Reductions of Microglial Activation at Posttreatment Follow‐up Monitoring (A) The figure shows delta reduction maps for the 3 patients who had a second _11_CPK11195 PET scan at posttreatment follow‐up monitoring. Delta reductions are expressed as the difference between _11_C‐PK11195 PET binding potential peaks (BP‐peaks) of microglial activation at baseline and BP‐peaks at follow‐up, computed voxel‐vise. The colored scale represents the BPpeaks decrease at follow‐up. (B) The figure shows longitudinal variation of the mean BPs values of each region of interest (ROI), i.e. ARIA‐E_roi (red columns) and ARWMC_roi (green columns), calculated at baseline (left side) and at posttreatment follow‐up monitoring (right side) for each patient. Mean BP values are expressed as BPs x 10_2_ for illustration purposes. Red circles represent BGTS values at each time point. ARIA‐E = amyloid‐related imaging abnormalities suggestive of vasogenic edema or sulcal effusion; ARWMC = age‐related white matter changes; BGTS = Barkhof Grand Total score; ROI = region of interest.

Ten weeks later, ^11^C-PK11195 PET revealed a globally reduced, although persistent, microglial activation (Figure 1), with more evident BP peak reduction in the ARIA-E anatomic region (Figure 3). BP mean values in ARIA-E_roi decreased from 0.49 ± 0.17 at baseline to 0.30 ± 0.14 at follow-up (−38%). BP mean values in ARMWC_roi decreased from 0.29 ± 0.13 to 0.19 ± 0.11 (−34%). CSF testing at follow-up was not performed, as a second lumbar puncture was not possible.

### Case 2

A 74-year-old woman was admitted with pharmacoresistant headache, behavioral changes, and rapid cognitive decline. The patient had a medical history of arterial hypertension, dyslipidemia, breast cancer with negative oncologic follow-up, and a history of possible CAA.

**Figure 4:**
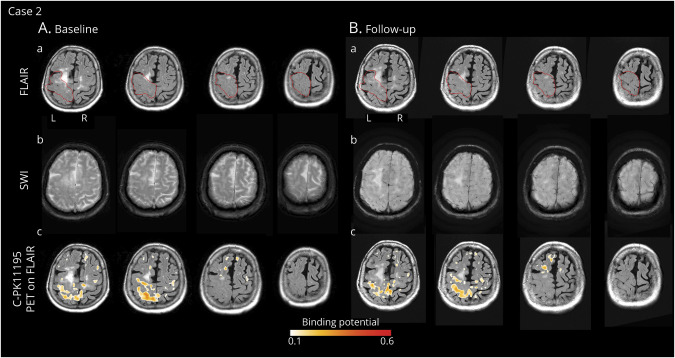
Longitudinal In Vivo Imaging of Microglial Activation During the Course of Spontaneous ARIA-E in a Patient With Possible CAA-ri (Case 2) (A) Baseline MRI and ^11^C-PK11195 PET images acquired 2 months after symptom onset. (a) T2-weighted (FLAIR) images showed spontaneous ARIA-E, i.e., reduced amplitude of sulci in the left superior parietal and precentral areas consistent with vasogenic edema (red lines indicate the anatomic regions affected by ARIA-E). (b) Gradient echo-T2*-weighted imaging (GRE-T2*) sequence, coregistered to baseline FLAIR images, showed multiple cerebral microbleeds (CMBs) in the right and left parietal lobes. CSF testing for anti-Aβ autoantibody was positive.^2^ The diagnosis of possible CAA-ri was made.^6^ (c) ^11^C-PK11195 PET images, coregistered and superimposed onto baseline FLAIR images, revealed clusters of microglial activation mainly localized within the ARIA-E region. Treatment with high-dose corticosteroid pulse therapy was started. One week later, second line therapy with oral azathioprine was started due to clinical worsening attributed to a new inflammatory flare. (B) Follow-up MRI and ^11^C-PK11195 PET images acquired 2.5 months from starting immunosuppressive therapy. MRI and PET images are coregistered to baseline FLAIR. (a) FLAIR images showed only a partial reduction of vasogenic edema within the ARIA-E region identified at baseline (red lines). (b) GRE-T2* images showed 1 new CMB, not localized within the ARIA-E region. CSF testing for anti-Aβ autoantibodies confirmed reduced levels compared with baseline.^2^ (c) ^11^C-PK11195 PET revealed a global reduced microglial activation that was more evident in the ARIA-E region. ARIA-E = amyloid-related imaging abnormalities suggestive of vasogenic edema; CAA-ri = cerebral amyloid angiopathy–related inflammation; FLAIR = fluid-attenuated inversion recovery.

Two months after the onset of symptom (delay due to an arm fracture), FLAIR-MRI images (Figure 4) revealed moderate ARIA-E in the left rolandic sulcus and parietal lobe with decreased sulcal spaces in the same area, with a BGTS value of 4. T2-GRE* images showed multiple CMBs in the left and right parietal and temporal lobes, corresponding to a CAA severity score of 2. The CSF testing for anti-Aβ autoantibodies was positive.^2^ The AT(N) biomarker profile resulted A+T+(N)−, suggesting AD pathologic changes. APOE genotyping showed ε2/ε4 allele carriage. All results are shown in the Table.

According to clinical presentation, MRIs, and CSF findings, a diagnosis of possible CAA-ri was made.^2^ One week after the MRI, ^11^C-PK11195 PET revealed BP peaks of microglial activation mainly localized within the ARIA-E anatomic region (Figure 4). The spatial interaction of BP peaks with ARIA-E_roi and ARWMC_roi was 3.7% and 1.9%, respectively (Figure 2).

Two weeks after PET, treatment with 5 IV boluses of 1 g/d methylprednisolone for 5 consecutive days was started. One week later, the patient presented with subacute left hemiparesis, compatible with a new inflammatory flare of CAA-ri, and immunosuppressive therapy with oral azathioprine was started, with a progressive improvement of neurologic symptoms. Three and a half months after admission (2.5 months after starting azathioprine), a second MRI showed a partial resolution of ARIA-E, with a 1-point reduction in the BGTS and mild radiologic severity. T2*-GRE images showed 1 new CMB that was not localized within the ARIA-E anatomic region.

**Figure 5:**
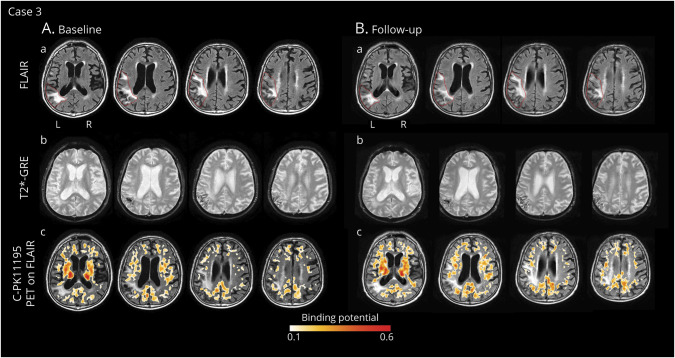
Longitudinal In Vivo Imaging of Microglial Activation During the Course of Spontaneous ARIA in a Patient With Possible CAA-ri (Case 3) (A) Baseline MRI and ^11^C-PK11195 PET images acquired 2.5 months after presentation of (sub)acute symptoms. (a) T2-weighted (FLAIR) images showed spontaneous ARIA-E in the left lateral fronto-temporo-parietal area (red lines indicate the anatomic regions affected by ARIA-E). (b) Gradient echo-T2*-weighted imaging (GRE-T2*) sequence, coregistered to baseline FLAIR images, showed disseminated cortical superficial siderosis in the left and right parietal lobes. CSF testing confirmed high concentrations of anti-Aβ autoantibodies.^2^ A diagnosis of possible CAA-ri was made.^6^ (c) 11C-PK11195 PET binding potentials (BP peaks), coregistered and superimposed onto baseline FLAIR, revealed scattered clusters of microglial activation only partially localized within the ARIA-E anatomic region. Treatment with high-dose corticosteroid pulse therapy was started, with progressive resolution of clinical symptoms. (B) Follow-up MRI and ^11^C-PK11195 PET acquired three and a half months after starting corticosteroids therapy. All images are coregistered to baseline FLAIR. (a) FLAIR images showed only a slight decrease in microglial activation, compared with baseline. (b) GRE-T2* images did not reveal any new microbleeds. CSF testing for anti-Aβ autoantibodies confirmed reduced levels compared with baseline.^2^ (c) ^11^C-PK11195 PET revealed a slight decrease in microglial activation within and outside the ARIA-E anatomic region. ARIA-E = amyloid-related imaging abnormalities suggestive of vasogenic edema; CAA-ri = cerebral amyloid angiopathy–related inflammation; FLAIR = fluid-attenuated inversion recovery.

One month and a half after the second MRI, CSF testing for anti-Aβ autoantibodies proved to be borderline, suggesting a noncomplete biological resolution.^2^ The AT(N) biomarker profile resulted A−T+(N)+, with markedly reduced levels of Aβ40 compared with baseline. Three months later, (7 months after starting azathioprine), ^11^C-PK11195 PET showed an overall decreased, although persistent, microglial activation (Figure 5), with a more evident BP peak reduction in the ARIA-E anatomic region (Figure 3).

Mean BP values in ARIA-E_roi decreased from 0.30 ± 0.10 at baseline to 0.22 ± 0.12 at follow-up (−26%). Mean BP values in ARMWC_roi decreased from 0.22 ± 0.10 to 0.18 ± 0.12 (−17%).

### Case 3

An 85-year-old man was admitted with a headache and altered mental state. The patient had a medical history of TIA in antiplatelet treatment and left parietal subarachnoid hemorrhage followed by left parietal cortical-subcortical ICH, respectively, 8 and 9 years, before admission.

A first CT scan at admission excluded an acute hemorrhage, and the patient was discharged. One month and a half later, the patient was readmitted due to persistence of symptoms, and the MRI scan (Figure 5) revealed mild ARIA-E in the left fronto-temporo-parietal area, with a BGTS value of 4. The T2*-GRE images showed disseminated cSS, bilaterally, consistent with a CAA severity score of 3.

The CSF testing for anti-Aβ autoantibodies was positive.^2^ The CSF sample was not sufficient to measure AT(N) biomarkers. ApoE genotyping showed ε3/ε4 allele carriage. All results are shown in Table.

According to clinical presentation, MRIs, and CSF findings, a diagnosis of possible CAA-ri was made.^2^ One month after MRI, ^11^C-PK11195 PET revealed significant but scattered BP peaks of diffuse microglial activation that was, however, only partially localized within the ARIA-E anatomic region (Figure 5). The spatial interaction of BP peaks with ARIA-E_roi and ARWMC_roi was 0.9% and 0.8%, respectively (Figure 2).

Three weeks later, treatment with IV high-dose corticosteroid pulse therapy, without oral tapering, was started with a progressive resolution of the clinical symptoms. Six months after admission (2.5 months after starting corticosteroids), a second MRI showed almost unchanged findings compared with baseline (Figure 5) and no change of both BGTS and ARIA-E severity scores. T2*-GRE images showed no new CMBs.

One month after the MRI, CSF testing for anti-Aβ autoantibodies was negative.^2^ The AT(N) biomarker profile resulted A−T+(N)+, suggesting non-AD pathologic changes. Two months and a half after the second MRI (5 months after starting corticosteroids), ^11^C-PK11195 PET showed a slight decrease in microglial activation (Figure 5) both globally and in the ARIA-E anatomic region (Figure 3).

Mean BP values in ARIA-E_roi decreased from 0.23 ± 0.08 at baseline to 0.15 ± 0.10 at follow-up (−33%). Mean BP values in ARMWC_roi decreased from 0.19 ± 0.08 to 0.16 ± 0.10 (−13%).

### Case 4

A 76-year-old woman was admitted with gait unsteadiness, headache, agitation, and confusion.^25^ The patient had a medical history of systemic arterial hypertension, mild depression, and amnestic mild cognitive impairment.

**Figure 6:**
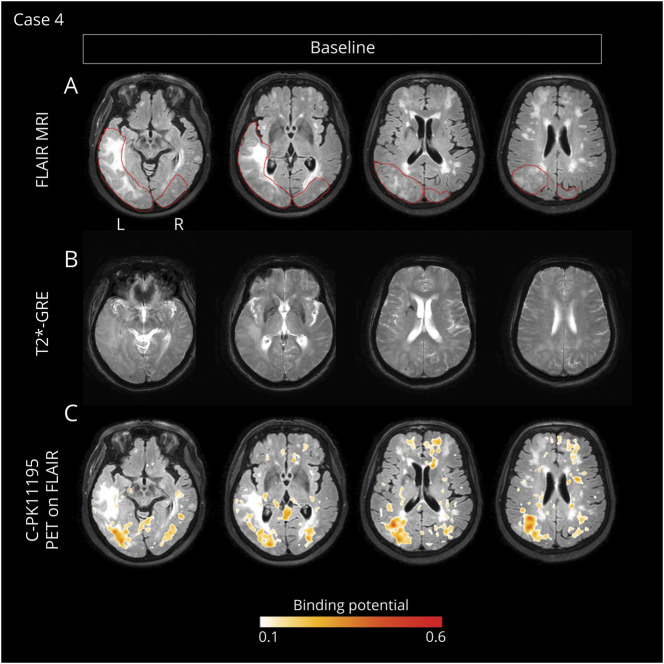
Longitudinal In Vivo Imaging of Microglial Activation During the Course of Spontaneous ARIA in a Patient With Possible CAA-ri (Case 4) Baseline MRIs and ^11^C-PK11195 PET acquired 3 weeks after symptom onset. (A) T2-weighted (FLAIR) images showed spontaneous ARIA-E in the left and right occipital lobes (red lines indicate the anatomic regions affected by ARIA-E). (B) Gradient echo-T2*-weighted imaging (GRE-T2*) sequence, coregistered to baseline FLAIR, showed disseminated cortical and subcortical microbleeds in the left temporal lobe and subarachnoid hemorrhage in the left parietal lobe. CSF testing confirmed high concentrations of anti-Aβ autoantibodies.^2^ A diagnosis of probable CAA-ri was made.^6^ (C) ^11^C-PK11195 PET binding potential peaks (BP peaks), coregistered and superimposed onto baseline FLAIR, reveal diffuse BP peak clusters of microglial activation that were mainly colocalized within the ARIA-E anatomic regions. The patient was treated with high-dose corticosteroid pulse therapy, with marked improvement of clinical symptoms. ARIA-E = amyloid-related imaging abnormalities suggestive of vasogenic edema; CAA-ri = cerebral amyloid angiopathy–related inflammation; FLAIR = fluid-attenuated inversion recovery.

Two weeks after symptom onset, the MRI (Figure 6) showed severe ARIA-E in the left and right occipital lobes, with a BGTS value of 7. T2*-GRE showed cortical-subcortical CMBs in the left temporal lobe along with a convexity subacute subarachnoid hemorrhage in the left parietal lobe. The patient received a CAA severity score of 4.

Two weeks later, CSF testing for anti-Aβ autoantibodies was positive.^2^ The AT(N) biomarker profile resulted A+T+(N)−, suggesting AD pathologic changes in the AD continuum. ApoE genotyping showed ε3/ε4 allele carriage. All results are shown in Table.

According to clinical presentation, MRIs, and CSF findings, a diagnosis of probable CAA-ri was made.^2^ One week after MRI, ^11^C-PK11195 PET revealed diffuse BP peaks of microglial activation that were more evident within the ARIA-E anatomic regions (Figure 6). The spatial interaction of BP peaks with ARIA-E_roi and ARWMC_roi was 27.0% and 9.0%, respectively (Figure 2).

Mean BP values in ARIA-E_roi and in ARMWC_roi were 0.32 ± 0.11 and 0.20 ± 0.08, respectively. The day after, treatment with IV high-dose corticosteroid pulse therapy was started, followed by 1 mg/kg of oral prednisolone and gradual tapering-off, with marked clinical improvement.

Four months after admission (3.5 months after starting corticosteroids), a second MRI showed nearly complete resolution of ARIA-E, with a 6-point reduction in the BGTS value. Further assessments were not possible as the patients refused to continue the study.

## Discussion

The exact mechanisms of ARIA-E remain to be fully elucidated. The review of lessons learned from the last decade of research confirmed the ARIA Paradox as the most accredited pathophysiologic model to explain the biological complexity of ARIA-E.^1,4,5,10,12,42^ According to this model, ARIA-E is a complex and multifactorial phenomenon resulting from the imbalance between the removal of Aβ deposited in plaques, which is attributed to the dose, time, and type of anti-Aβ (auto)antibodies, and the downstream effects that an excessive mobilization of Aβ can cause on intramural periarterial drainage pathways,^43^ which may account for increased transient CAA, cerebrovascular impairment, greater vascular permeability, and an easier extravasation of proteinaceous fluid and VE, particularly in ApoE ε4 carriers.^1,2,10,12,44–46^

In this proof-of-principle study, we extend the understanding of ARIA-E biology by providing in vivo evidence for an association between ARIA-E and microglial activation through multimodal and multiparametric MRI, CSF testing for anti-Aβ autoantibodies and AT(N) biomarkers, and ^11^C-PK11195 PET longitudinal assessments in patients with CAA-ri, a spontaneous human model of the iatrogenic ARIA-E of AD immunotherapy.^1,2,10,12,15,16^ At (sub)acute presentation of ARIA-E, we observed scattered and diffused clusters of ^11^C-PK11195 PET BPs that, although heterogeneously distributed across subjects, were mainly localized in the posterior brain regions. At a single-subject level, the patterns of increased microglial activation peaks showed a greater spatial interaction with ARIA-E than with chronic ARWMC of cSVD and aging, which was consistent in all participants (mean % overlap for ARIA-E 16% vs 4% for ARWMC). Together, these data suggest a specific association between the (sub)acute presentation of ARIA-E and the focal increase of microglial activation.

Our results also showed a clearly more marked spatial colocalization of ARIA-E with the focal clusters of increased microglial activation peaks in probable CAA-ri compared with possible CAA-ri, the latter showing a 10 times lesser overlap (on average 29% vs 2.3%, respectively). Probable CAA-ri also had the highest ARIA-E radiographic severity^28^ and BGTS^27^ values. Considering that CAA-ri criteria have a sensitivity of 82% and a specificity 97% in diagnosing probable CAA-ri, while the specificity reduces to 68% for possible CAA-ri,^6^ one could speculate that some of our participants have been misdiagnosed. However, we believe that this hypothesis is unlikely. In fact, our participants underwent CSF testing for anti-Aβ autoantibody positivity,^2^ and all showed good response to corticosteroids, which is in keeping with the 82% of clinical-radiologic resolution of spontaneous ARIA-E following immunosuppressive therapy as recently reported by Antolini et al.^1^

An alternative explanation, as suggested by our data, is that the magnitude of microglial activation associated with ARIA-E could be mainly influenced by the coexistence of CAA and AD instead of the presence of one disease as a single entity. In fact, our results showed that the patients with copresence of both CAA and AD pathology, as measured with MRI and CSF biomarkers, also had (1) the highest ^11^C-PK11195 PET BP mean values; (2) the most severe ARIA-E radiologic manifestations; and (3) the highest BGTS values. Notably, the diagnosis of CAA-ri is not affected by the CAA burden, as the current criteria simply require the presence of ≥1 imaging marker of CAA to make both probable and possible diagnosis, irrespective of the number and type of bleeding manifestations.^6^ Notwithstanding, case 2 and case 3, which both had only CAA but no evidence of coexisting AD according to the AT(N) biomarker profile framework, showed the lowest microglial activation. The finding is even more interesting if we consider that, despite case 3 having a more severe CAA pathology compared with case 2, both had similar ARIA-E radiologic scores (i.e., mild/moderate ARIA-E severity and low BGTS values).

Together, our data provide a strong support to the hypothesis that ARIA-E is the expression of an increased overload of Aβ caused by an exaggerated therapeutic effect of (auto)antibodies in disassembling Aβ plaques and the related side effects that this overload can generate on the preexisting CAA.^1,2,10,12^ In this framework, our findings highlight that MRI rating scales alone may not fully interpret the focal nature of the complex underlying biology underlying ARIA-E and point out the need for additional CSF or plasma biomarkers to help reduce current heterogeneity in the interpretation of ARIAs.

At posttreatment monitoring, our results showed a marked 2.3-fold reduction of microglial activation associated with ARIA-E compared with ARWMC. This finding supports the potential therapeutic effectiveness of corticosteroids in the management of the neuroinflammatory processes associated with ARIA-E, which is in keeping with evidence from a large cohort of 110 patients with CAA-ri.^1^

Notably, we would highlight that although all patients showed a full clinical resolution of acute symptoms, none of them reached a full radiologic resolution of ARIA-E, as measured with current MRI rating systems (i.e., a BGTS = 0). This could be interpreted in various ways. First, it could be that more than 5 months is necessary for the complete resolution of the neuroinflammation associated with ARIA-E, which is in keeping with the drastic but noncompletely reduced microglial activation we observed in all but one patient, who was, however, the only one treated with azathioprine. Moreover, given the slight differences in the timing of PET acquisitions in respect to the first presentation of symptoms, further study is needed to exclude any temporal association of microglial activation with the symptomatic manifestation of ARIA-E. Second, given that current ARIA-E rating scales have not been validated for their use outside the setting of a clinical trial, real-world data from large cohorts of CAA-ri are needed to prove their applicability in clinical practice (i.e., patients with no pre-event MRIs for making proper comparisons and patients with moderate/severe underlying cSVD comorbidity). Nevertheless, we believe that this is a strength of our study, as it first provides a preliminary overview of the scenario we should expect to manage given the recent approval of aducanumab for its clinical use in the heterogeneous community of patients with AD. Considering that at least some degree of CAA is present in most patients with AD as well as older people, the burden of ARIA-E complications in the real clinical practice would be anticipated to potentially increase compared with the reported 43% incidence in the EMERGE and ENGAGE clinical trials with aducanumab.^1,10,12,20,28,42^ Then, elucidating the nature and the response to treatment course of the neuroinflammation associated with ARIA-E will be of paramount importance for both diagnostic and treatment decisions.^1^

In this framework, our findings give a strong support in the use of corticosteroids to manage the neuroinflammatory response mediated by increased CSF anti-Aβ (auto)antibodies^1,3^ and highlight the urgency to define specific treatment and monitoring recommendations for ARIA-E.^1,10,12^ Indeed, CSF anti-Aβ autoantibodies were elevated at disease presentation and decreased at follow-up when cerebral microglial activation was reduced. This is in keeping with the hypothesis that immunotherapy-induced ARIA-E are iatrogenic manifestations of the spontaneously occurring ARIA-E of CAA-ri and point to CSF testing for anti-Aβ (auto)antibodies as a promising diagnostic, prognostic, and response to treatment biomarker of ARIA-E.^2,10,12,47,48^

Further research to validate the utility of the biomarker is needed to reduce current heterogeneity in the interpretation of trials' results and improve the detection, therapeutic management, and monitoring of ARIA-E side events.^8,^^49,^^50^

In this context, our results are of paramount importance for expediting the design of future confirmatory studies, e.g., (1) the need of testing patients within the first month from ARIA-E diagnosis, as the rate of microglial activation may change very quickly; (2) the importance of carefully considering preexisting CAA in addition to the Aβ plaque burden; (3) the potential of CSF testing for anti-Aβ (auto)antibodies; and (4) the urgent need of randomized clinical trials to confirm the effectiveness of corticosteroids in the prevention of ARIA-E.^1^

Filling these knowledge gaps is of paramount importance if we consider that current guidelines suggest an empirical cutoff value of 5 microhemorrhages for patient enrollment in clinical trials and that no data exist on primary prevention with mAbs in patients with CAA. If the ARIA Paradox is true, it is possible that patients with CAA, but no AD copathology, have a reduced risk of ARIA-E. This is of paramount importance as it may open to new therapeutic horizons also for this orphan disease, including reconsideration of current inclusion and exclusion recommendations in AD immunotherapy.

The main strength of our study is the providing of critical in vivo evidence for a temporal and regional association of microglial activation with ARIA-E and high CSF levels of anti-Aβ autoantibodies in the context of coexisting CAA and AD pathology. Our study also has several limitations. First, although the accurate characterization of our patients, including the use of gold standard and state-of-the-art assessments for ARIA-E, anti-Aβ autoantibody dosage, and CAA, we would like to stress that the low number of cases and the heterogeneous nature of ARIA-E manifestations prevented any statistical analyses. As such, our findings should not be interpreted outside the intention of a proof-of-concept study aimed to first verify the presumed association of ARIA-E and microglial activation, as suggested by few ex vivo studies^17–19^ and as hypothesized in the ARIA Paradox model.^1,2,10,12^ Second, as only patients who were clinically stable and able to travel were included in this study, we cannot exclude our findings restrict to less severe presentations and thus do not represent the whole picture of such a heterogeneous and complex condition. Third, amyloid-PET imaging and neuropathology data were not available, and the longitudinal CSF testing for AT(N) biomarkers was available only for one case. This precluded any further analysis on the dynamic change variations of Aβ associated with an ARIA-E event, such as the specific contribution of microglial activation in the focal removal of parenchymal Aβ vs an increased vascular deposition of Aβ in the form of CAA.

## Glossary

AD: Alzheimer disease
ARIA-E: amyloid-related imaging abnormalities suggestive of vasogenic edema
ARWMC: age-related white matter change
Aβ: β-amyloid
BGTS: Barkhof Grand Total Score
BP: binding potential
CAA-ri: cerebral amyloid angiopathy–related inflammation
CMB: cerebral microbleed
cSS: cortical superficial siderosis
cSVD: cerebral small vessel disease
FLAIR: fluid-attenuated inversion recovery
GRE-T2*: gradient echo-T2* weighted
ICH: intracerebral hemorrhage
mAb: monoclonal antibody
QAlb: CSF/serum albumin quotient
QNorm: normal CSF/serum albumin
ROI: region of interest
TAC: time-activity curve
UNIMIB: University of Milano-Bicocca
VE: vasogenic edema
